# A major entomoparasite interferes with the chikungunya virus transmission by *Aedes albopictus*

**DOI:** 10.1002/mlf2.70021

**Published:** 2025-06-23

**Authors:** Edwige Martin, An‐nah Chanfi, Barbara Viginier, Vincent Raquin, Claire Valiente Moro, Guillaume Minard

**Affiliations:** ^1^ Universite Claude Bernard Lyon 1, CNRS, INRAE, VetAgro Sup, UMR Ecologie Microbienne Villeurbanne France; ^2^ IVPC UMR754, EPHE, PSL Research University, INRAE, Univesite Claude Bernard Lyon 1 Lyon France

## Abstract

The Asian tiger mosquito, *Aedes albopictus*, is an invasive species that spreads diseases like chikungunya and has caused outbreaks worldwide. Studies show that mosquito‐associated microbes can affect disease transmission. One of those microbes, the parasite *Ascogregarina taiwanensis*, is common in native and settled mosquito populations (>3 years after introduction) but rare in recently introduced ones. We found that this parasite slows down the spread of the chikungunya virus within the mosquito and decreases its transmission rate by half. Unparasitized mosquitoes spread the virus more easily, suggesting that changes in mosquito‐associated microbes could impact disease outbreaks and public health.

The chikungunya virus (CHIKV) is an arthropod‐borne virus (arbovirus) causing febrile illness, a rash, and severe and often debilitating arthralgias that may last for several years in humans. Originally, it was mostly vectored by the Yellow fever mosquito *Aedes aegypti* but, due to a single mutation (i.e., E1‐A226V), its vector specificity has shifted toward an efficient transmission by the Asian tiger mosquito *Aedes albopictus* ^1^, ^2^. The two mosquito species are now considered as major vectors of the virus.

Originating from southern and eastern Asia, *Ae. albopictus* is the most invasive mosquito species and has recently been introduced in every inhabited continent due to global trade^3^. The combined impact of mosquito invasiveness and circulation of CHIKV strains carrying the E1‐A226V mutation has drastically promoted the emergence of explosive chikungunya outbreaks. The Indian Ocean, in particular, was strongly impacted between 2004 and 2007, with one‐third of the population from La Réunion island being infected and 10% of the patients presenting disabling after‐effects 13 years later^4^. To be transmitted from one individual to another, the virus needs to be acquired by a competent female mosquito when biting an infected host. Following this natural entry route, the virus replicates in the mosquito midgut epithelium to get disseminated throughout the mosquito body and reach the salivary glands. CHIKV particles are then transmitted to a healthy host through saliva that is secreted during a blood meal.

During the last decades, many studies have suggested that pathogen transmission can be modulated by mosquito‐associated microorganisms forming their microbiota^5^. Some microorganisms interfere with the mosquito vector competence that is defined by their ability to efficiently get infected, replicate, and retransmit a given pathogen^6^. The microbiota of a given mosquito can (i) directly interfere with pathogens, for example, by lysing them^7^ or (ii) indirectly interfere with pathogens by reinforcing the insect's natural barrier such as their innate immunity^8^. However, we are still lacking information on the consequences of variations in the mosquito microbiota toward pathogen transmission. Prokaryotic and eukaryotic microbiota in *Ae. albopictus* have been reported to vary among populations, presumably due to environmental conditions and population dynamics^9^. If all the populations and almost all the individuals are infected by the dominant intracellular bacteria *Wolbachia w*AlbA and *w*AlbB, they show marked variations in their association with the second most dominant microorganism, the extracellular apicomplexan specialized entomoparasite *Ascogregarina taiwanensis*.

These variations are directly related to the global spread of *Ae. albopictus*. Indeed, *As. taiwanensis* colonizes more than 80% of individuals in native or settled mosquito populations (i.e., >3 years after introduction), while it colonizes less than 20% of the individuals in recently introduced ones (i.e., <3 years after introduction), presumably due to its weak ability to survive under mosquito egg transportation conditions^9^. This protist is considered as a weak mosquito parasite colonizing the adult Malpighian tubules and that is only costly for its host when conditions are harsh^10^. Preliminary studies evidenced that the presence of *As. taiwanensis* reduces the density of the pathogenic dog earthworm *Dirofilaria immitis* in *Ae. albopictus* ^11^. In this study, we proposed to investigate its effect on *Ae. albopictus* vector competence toward CHIKV. To do so, we compared the dynamic of infection, dissemination, and transmission of the virus over time after unparasitized and parasitized mosquitoes have been fed with an infectious blood meal (Figure 1A). Parasitized and unparasitized mosquito strains were generated from a mosquito population that has been collected in Villeurbanne and Pierre‐Bénite (France) in 2017 and present natural infection of *As. taiwanensis* (see the Supporting Information). Further information about the generation and maintenance of mosquito strains has been previously detailed^12^. The experiment was repeated twice on two mosquito generations. We have then correlated the viral and parasite (i.e.*,* oocyst form) load in coinfected individuals. Details on the methods are available in the Supporting Information.

**Figure 1:**
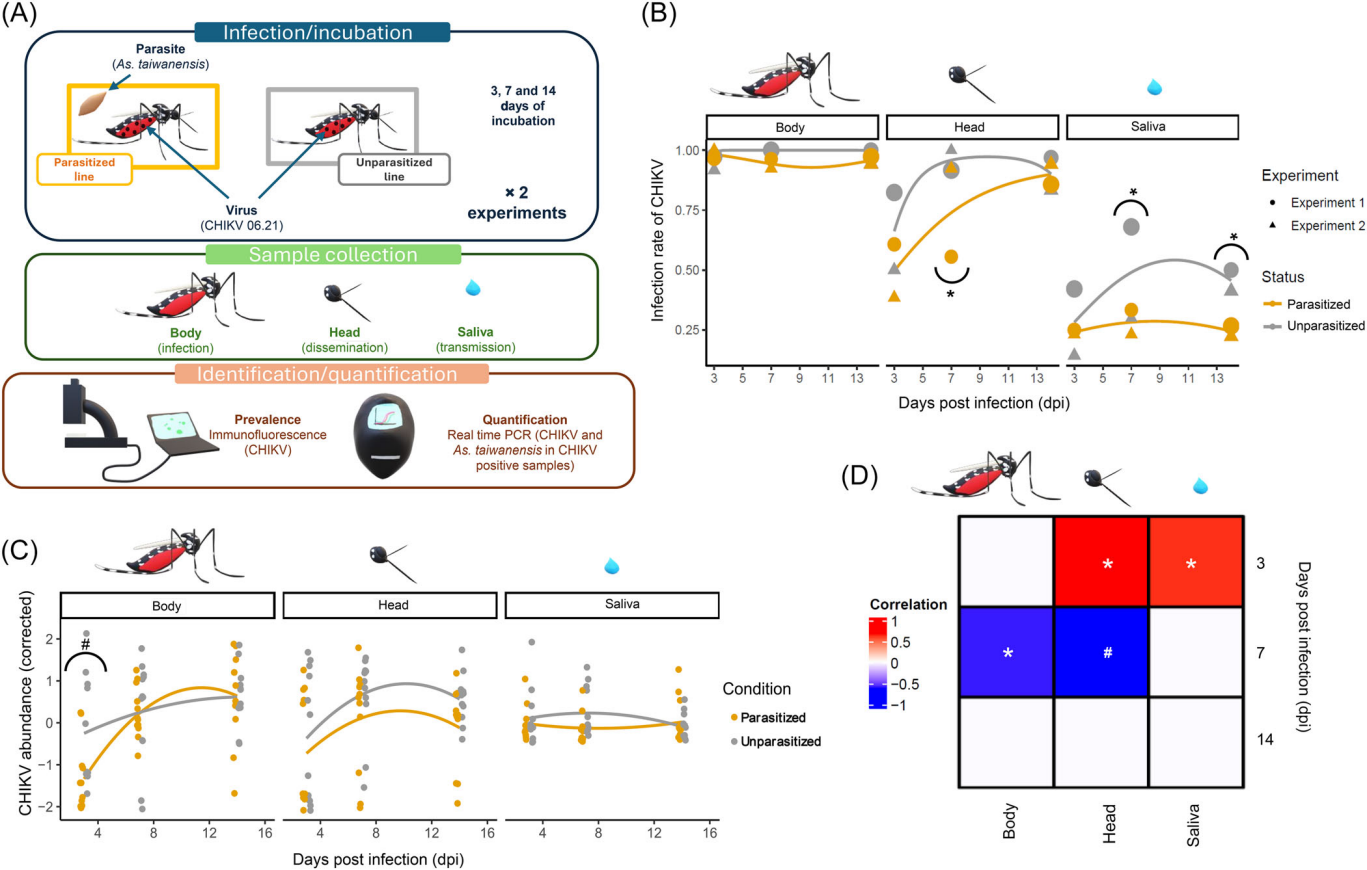
Impact of *Ascogregarina taiwanensis* parasitism on *Aedes albopictus* vector competence for chikungunya virus (CHIKV). (A) Scheme of the experimental design. Parasitized and unparasitized mosquitoes were fed a blood meal containing CHIKV strain 06.21 (~10⁷ FFU/ml). The experiment was repeated twice on different mosquito generations. For each experiment and each time point (3, 7, or 14 days post infection (dpi)), 10 to 38 engorged females were sampled. Mosquito bodies, heads, and saliva were collected, and CHIKV presence was assessed by immunofluorescence on C6/36 cell lines. A subset of 5–6 CHIKV‐positive individuals (per sample type, time point, and experiment) was further analyzed by RT‐qPCR to quantify both the virus and the parasite. (B) Infection rate of CHIKV in mosquitoes over time. CHIKV prevalence (infection rate) is shown for each sample type and time point across the two experiments. Dot size indicates sample size (10–38 mosquitoes). Lines represent second‐order polynomial regressions of prevalence over time. Parasitized mosquitoes are shown in orange; unparasitized mosquitoes are shown in gray. (C) CHIKV abundance in mosquitoes over time. CHIKV relative abundances are shown after correcting for inter‐experiment variations. Lines show third‐order polynomial regressions over time, with colors as in (B). (D) Correlations between the abundance of *As. taiwanensis* and CHIKV load. For mosquitoes co‐infected with *As. taiwanensis* and CHIKV, correlations between parasite and virus loads are shown by time point (rows) and sample type (columns). Positive Spearman correlations appear in red and negative Spearman correlations appear in blue. Significance/tendency levels from Tukey HSD *post hoc* tests (for prevalence and abundance, B and C) or Spearman correlations (D) are indicated: *p < 0.05; ^#^ *p* < 0.1.

Prevalence of the virus was estimated by immunofluorescence after infecting on C6/36 cells with crushed body, head, or saliva samples from parasitized and unparasitized mosquitoes 3, 7, and 14 days after being fed with an infectious blood meal (i.e., days post infection, dpi). The virus infection rate was measured as the proportion of positive mosquito bodies for the virus presence. It reached almost 100% and it was not influenced by the mosquito parasitic status and was stable over time (Figure 1B; Table S1). Conversely, the proportion of mosquitoes with virus dissemination to the head increased over time, regardless of parasitic status (Table S1), although this dissemination was slightly delayed in parasitized individuals. Indeed, at 7 dpi, unparasitized mosquitoes were 6.74 ± 4.63 times more likely to harbor disseminated viruses in mosquito heads than their parasitized conspecifics (Figure 1B). Although the difference was not significant at other time points, with on average 64% ± 0.05% of infected mosquito heads at 3 dpi and 90.2% ± 0.03% at 14 dpi (Figure 1B). The proportion of females being able to transmit CHIKV via saliva did not significantly differ over time (probably due to a lack of statistical power), while it was significantly influenced by the parasitic status of mosquitoes (Table S1). In more detail, the proportion of mosquitoes that released CHIKV via saliva at 3 dpi was relatively similar between both parasitized and unparasitized status, while it was lower for parasitized individuals with 3.13 ± 1.6 and 2.66 ± 1.19 times less chance to transmit viral particles at 7 and 14 dpi (Figure 1B).

Viral RNA load was estimated by RT‐qPCR of the E2 virus gene and normalized by the mosquito RPS17 housekeeping gene (for mosquito tissues) for five to six samples that were previously identified as infected by the virus and for each experiment, each time point, and each sample. The viral load increased over time in the body and head of mosquitoes, regardless of their parasitic status type (Figure 1C). We also observed a slight tendency for an interaction between the mosquito condition and days post‐infection. Indeed, CHIKV abundances were slightly lower in the body of parasitized mosquitoes compared to their unparasitized relatives at an early time point, while no significant difference was observed in the later ones. Also, no differences were observed in CHIKV abundances between parasitized and unparasitized mosquitoes in the head and saliva (Figure 1C; Table S1).

When individuals were simultaneously infected by the parasite and the virus, we also checked whether the parasite density correlates with the viral load that infects the mosquito, disseminates into its head, or is transmitted through saliva over time. At 3 dpi, abundances of parasites were positively correlated with viral load in the mosquito head and saliva (Figure 1D; Table S2 **)**. At 7 dpi, the trend tends to differ. The parasite abundance is negatively correlated with the viral load in the mosquito body and a similar tendency was shown for the head, while no significant correlation was observed in the mosquito saliva. Finally, no correlation was observed between the parasite and CHIKV abundances in the body, head, and saliva of the mosquito at 14 dpi.

Here, we provide the first proof‐of‐concept results showing that *Ascogregarina* parasites alter the vector competence of *Ae. albopictus* toward CHIKV. To do this, we used a mosquito population for which we previously estimated CHIKV 06.21 intra‐vector dynamics for a range of human viremia‐like CHIKV doses^13^. The dynamic reported in that former study was very close to that of the non‐parasitized individuals in this current investigation. Therefore, deviation of dissemination and transmission in parasitized mosquitoes can be confidently inferred in terms of the interaction of individuals with *As. taiwanensis*. Until now, *Wolbachia* was the mosquito‐associated major microbial symbiont for which viral interference was intensively studied^14^. The bacterium was shown to block the transmission of multiple viruses. Given this property, transinfection of mosquitoes with pathogen‐blocking strains of *Wolbachia* has been proposed as a biocontrol tool to limit arbovirus spread. Mechanisms behind its antiviral activities remain elusive but interactions with cellular autophagy^15^, lipid metabolism (in particular cholesterol scavenging), oxidative stress, and innate immunity^16^ have been proposed. Since *Wolbachia w*AlbA and *w*AlbB are highly prevalent and abundant in *Ae. albopictus* ^17^, which is also efficiently transmitting CHIKV^13^, these strains are not likely to strongly interfere with the virus *in insecta*. However, the triple interaction with *As. taiwanensis* may result in a shift in the bacteria physiology or abundance, resulting in a negative interaction with the virus (Figure 1D).

It is important to note that the virus and the parasite are not colocalized in key organs for virus replication in adult females since the parasite colonizes Malpighian tubules during this life stage^18^, which are posterior to the midgut, where CHIKV replicates before its dissemination to salivary glands through the hemolymph. *As. taiwanensis*‐mediated modulation of the insect immune system has previously been observed^11^. This may regulate the host response to virions. Metabolic interactions may also occur. Indeed, a genome survey of the parasite suggests that it could contribute to the lipid metabolism of its host^19^ and so does the CHIKV^20^. We observed that parasite density was positively correlated with virus dissemination 3 dpi in head and saliva but negatively correlated with viral density in the body at 7 dpi, with no correlations detected at 14 dpi (Figure 1D). Since parasite density remains relatively stable throughout the experiment (Figure S1), this pattern may result from carryover effects of midgut damage caused by the parasite during early mosquito development, initially increasing permissiveness to viral dissemination. Indeed, the midgut barrier is a major bottleneck that filters out most of the virions^21^ and *As. taiwanensis* replicates within this same organ in the 2nd–4th larval instar^18^. At 7 dpi, negative correlations may result from a delayed competition for nutrition, metabolic, or immune responses triggered by high parasite densities, ultimately leading to aftereffects on the virus.

Previous studies showed that microbiota of *Ae. albopictus* is less diverse in recently introduced populations compared to native ones^9^, ^17^. This is partly due to the loss of this parasite, a dominant member of the native population's microbiota, during the transportation of mosquito eggs. As a result, recently introduced populations escape from their parasite during the first 2 years after introduction. Considering our knowledge on the distribution of this parasite, we suggest that its negative impact on mosquito vector competence could be considered in the near future as a proxy for improving epidemiological risk estimation. However, other factors that lead to variations in the transmission of CHIKV (e.g., other members of the microbiota, vector biting rate, survival, and density) should also be considered and integrated into such models^5^. Further experimental studies are needed to determine whether these factors interact with *As. taiwanensis* to influence the vectorial capacity of *Ae. albopictus* for CHIKV in the field. Biocontrol strategies relying on *As. taiwanensis* properties may also be investigated, since this is a specialized mosquito parasite that is not known to infect other arthropods or vertebrates^10^.

In conclusion, we provide the first evidence on the impact of a natural and prevalent member of the *Ae. albopictus* microbiota on one of the major arboviruses that it transmits. The interference effect of the *Apicomplexa As. taiwanensis* against CHIKV is a promising step toward the understanding of microbiota‐induced disparities in mosquito vector competence. We evidenced that parasitism of the mosquito delays the dissemination and decreases the transmission rate of CHIKV particles by infected mosquitoes under controlled conditions. Since recently introduced mosquito populations escaped from the parasite compared to settled and native ones, these results may help to better model the epidemiological risk related to CHIKV transmission over time after *Ae. albopictus* invade a new territory. However, further studies of *As. taiwanensis* on mosquito fitness (e.g., survival, reproduction) and behavior (e.g., biting rate) as well as its interaction with the rest of the microbiota and their underlying consequences for virus transmission may be required to better estimate the CHIKV transmission risk. Finally, a better understanding of these interactions may contribute to the development of biocontrol strategies based on the utilization of such microbial agents as already experimented in the field for the *Wolbachia* endosymbiont.

## AUTHOR CONTRIBUTIONS

**Edwige Martin**: Conceptualization; investigation; methodology; resources; validation; writing—review and editing. **An‐nah Chanfi**: Data curation; investigation; methodology; validation; writing—review and editing. **Barbara Viginier**: Investigation; methodology; validation; writing—review and editing. **Vincent Raquin**: Conceptualization; investigation; methodology; validation; writing—review and editing. **Claire Valiente Moro**: Conceptualization; investigation; methodology; project administration; resources; supervision; writing—review and editing. **Guillaume Minard**: Conceptualization; data curation; formal analysis; funding acquisition; investigation; methodology; project administration; resources; supervision; validation; visualization; writing—original draft; writing—review and editing.

## ETHICS STATEMENT

Parasitized and unparasitized female mosquitoes were fed on anesthetized mice during the amplification steps of the populations. This protocol was reviewed by the Institutional Animal Care and Use Committee, acceptance reference number: Apafis #31807‐2021052715018315. Use of human blood donors for the mosquito infectious blood meal was enabled by an agreement with the French National Blood Bank: CODECOH DC‐2019‐3507.

## CONFLICT OF INTERESTS

The authors declare no conflict of interests.

## Supporting information

Supporting information revision.

## Data Availability

Raw data are available on Zenodo. https://doi.org/10.5281/zenodo.15350994.

## References

[1] Bonizzoni M , Gasperi G , Chen X , James AA . The invasive mosquito species *Aedes albopictus*: current knowledge and future perspectives. Trends Parasitol. 2013;29:460–468. 23916878 10.1016/j.pt.2013.07.003 PMC3777778

[2] Coffey L , Failloux AB , Weaver S . Chikungunya virus–vector interactions. Viruses. 2014;6:4628–4663. 25421891 10.3390/v6114628 PMC4246241

[3] Goubert C , Minard G , Vieira C , Boulesteix M . Population genetics of the Asian tiger mosquito *Aedes albopictus*, an invasive vector of human diseases. Heredity. 2016;117:125–134. 27273325 10.1038/hdy.2016.35 PMC4981682

[4] Guillot X , Ribera A , Gasque P . Chikungunya‐induced arthritis in Reunion Island: a long‐term observational follow‐up study showing frequently persistent joint symptoms, some cases of persistent chikungunya immunoglobulin M positivity, and no anticyclic citrullinated peptide seroconversion after 13 years. J Infect Dis. 2020;222:1740–1744. 32428203 10.1093/infdis/jiaa261

[5] Cansado‐Utrilla C , Zhao SY , McCall PJ , Coon KL , Hughes GL . The microbiome and mosquito vectorial capacity: rich potential for discovery and translation. Microbiome. 2021;9:111. 34006334 10.1186/s40168-021-01073-2 PMC8132434

[6] Zhu Y , Cao Y , Jiang L , Wang P , Cheng G . Interactions between commensal microbes and mosquito‐borne viruses. Annu Rev Virol. 2025;12:1.1–1.14. 10.1146/annurev-virology-092623-101222 40067963

[7] Ramirez JL , Short SM , Bahia AC , Saraiva RG , Dong Y , Kang S , et al. *Chromobacterium* Csp_P reduces malaria and dengue infection in vector mosquitoes and Has entomopathogenic and in vitro anti‐pathogen activities. PLoS Pathog. 2014;10:e1004398. 25340821 10.1371/journal.ppat.1004398 PMC4207801

[8] Gendrin M , Turlure F , Rodgers FH , Cohuet A , Morlais I , Christophides GK . The peptidoglycan recognition proteins PGRPLA and PGRPLB regulate anopheles immunity to bacteria and affect infection by plasmodium. J Innate Immun. 2017;9:333–342. 28494453 10.1159/000452797 PMC5569699

[9] Girard M , Martin E , Vallon L , Tran Van V , Da Silva Carvalho C , Sack J , et al. Human‐aided dispersal and population bottlenecks facilitate parasitism escape in the most invasive mosquito species. PNAS Nexus. 2024;3:pgae175. 38715727 10.1093/pnasnexus/pgae175 PMC11074241

[10] Lantova L , Volf P . Mosquito and sand fly gregarines of the genus *Ascogregarina* and *Psychodiella* (Apicomplexa: Eugregarinorida, Aseptatorina)—overview of their taxonomy, life cycle, host specificity and pathogenicity. Infect Genet Evol. 2014;28:616–627. 24797386 10.1016/j.meegid.2014.04.021

[11] Comiskey NM , Lowrie RC , Wesson DM . Effect of nutrient levels and *Ascogregarina taiwanensis* (Apicomplexa: Lecudinidae) infections on the vector competence of *Aedes albopictus* (Diptera: Culicidae) for *Dirofilaria immitis* (Filarioidea: Onchocercidae). J Med Entomol. 1999;36:55–61. 10071493 10.1093/jmedent/36.1.55

[12] Martin E , Vallon L , Da Silva Carvalho C , Girard M , Minard G . Gregarine parasites are adapted to mosquito winter diapause. Parasit Vectors. 2022;15:249. 35820959 10.1186/s13071-022-05365-w PMC9277866

[13] Viginier B , Cappuccio L , Garnier C , Martin E , Maisse C , Valiente Moro C , et al. Chikungunya intra‐vector dynamics in *Aedes albopictus* from Lyon (France) upon exposure to a human viremia‐like dose range reveals vector barrier's permissiveness and supports local epidemic potential. Peer Commun J. 2023;3:e96.

[14] Minard G , Mavingui P , Moro CV . Diversity and function of bacterial microbiota in the mosquito holobiont. Parasit Vectors. 2013;6:146. 23688194 10.1186/1756-3305-6-146 PMC3667145

[15] Voronin D , Cook DAN , Steven A , Taylor MJ . Autophagy regulates Wolbachia populations across diverse symbiotic associations. Proc Natl Acad Sci USA. 2012;109:E1638–E1646. 22645363 10.1073/pnas.1203519109 PMC3382551

[16] Zheng R , Wang Q , Wu R , Paradkar PN , Hoffmann AA , Wang GH . Holobiont perspectives on tripartite interactions among microbiota, mosquitoes, and pathogens. ISME J. 2023;17:1143–1152. 37231184 10.1038/s41396-023-01436-7 PMC10356850

[17] Minard G , Tran FH , Van VT , Goubert C , Bellet C , Lambert G , et al. French invasive Asian tiger mosquito populations harbor reduced bacterial microbiota and genetic diversity compared to Vietnamese autochthonous relatives. Front Microbiol. 2015;6:e970. 10.3389/fmicb.2015.00970 PMC4585046 26441903

[18] Chen WJ . The life cycle of *Ascogregarina taiwanensis* (Apicomplexa:Lecudinidae). Parasitol Today. 1999;15:153–156. 10322337 10.1016/s0169-4758(99)01418-0

[19] Templeton TJ , Enomoto S , Chen WJ , Huang CG , Lancto CA , Abrahamsen MS , et al. A genome‐sequence survey for *Ascogregarina taiwanensis* supports evolutionary affiliation but metabolic diversity between a Gregarine and Cryptosporidium. Mol Biol Evol. 2010;27:235–248. 19778951 10.1093/molbev/msp226 PMC2877549

[20] Melendez‐Villanueva MA , Trejo‐Ávila LM , Galán‐Huerta KA , Rivas‐Estilla AM . Lipids fluctuations in mosquitoes upon arboviral infections. J Vector Borne Dis. 2021;58:12–17. 34818858 10.4103/0972-9062.313961

[21] Forrester N , Coffey L , Weaver S . Arboviral bottlenecks and challenges to maintaining diversity and fitness during mosquito transmission. Viruses. 2014;6:3991–4004. 25341663 10.3390/v6103991 PMC4213574

