# Supplementary material for: A major entomoparasite interferes with the chikungunya virus transmission by Aedes albopictus
Source: mLife. 2025 Jun 23;4(3):337–41. doi: 10.1002/mlf2.70021 (PMC12207900; doi:10.1002/mlf2.70021)
Supplement: Supplementary file 1 — Supporting information revision. [file MLF2-4-337-s001.docx]

**Supporting information**

**A major entomoparasite interferes with chikungunya virus transmission by *Aedes albopictus***

**Edwige Martin^1^, An-nah Chanfi^1^, Barbara Viginier^2^, Vincent Raquin^2^, Claire Valiente Moro^1^, Guillaume Minard^1*^**

^1^Universite Claude Bernard Lyon 1, CNRS, INRAE, VetAgro Sup, UMR Ecologie Microbienne, F-69622 Villeurbanne, France.

^2^IVPC UMR754, EPHE, PSL Research University, INRAE, Universite Claude Bernard Lyon 1, F-69007 Lyon, France.

**Materials and methods**

Mosquito lines

Parasitized and unparasitized mosquito strains were generated from a mosquito population that has been collected in Villeurbanne (N: 45°46’18990’’ E: 4°53’24615’’) and Pierre-Bénite (N: 45°42’11534’’ E: 4°49’28743’’) in France in 2017 and presenting natural infection of *Ascogregarina taiwanensis.* South-west European populations present a mixed pattern of infection with the parasite due to their recent invasion history (1). Meanwhile, they have been recently involved in Chikungunya outbreaks (2–4). Therefore, those populations are relevant regarding the triple interaction (CHIKV - *As. taiwanensis* - *Ae. albopictus*) tested in this study. Further information on mosquito strains generation and maintenance were previously detailed (5). We verified the presence and absence of the parasite in each strain by microscopy and diagnostic qPCR (see protocol details in the next sections).

Mosquito exposure to Chikungunya virus by artificial blood meal

Experiments were conducted in level 3 biosafety facility (BSL3). Mosquitoes were exposed to CHIKV 06.21 (provided by Anna-Bella Failloux, Paris Pasteur Institute) originating from La Réunion Outbreak (2005-2006) and carrying the E1-A226V mutation. Previous experiments showed that the mosquito population was highly competent toward this viral strain (6). Virus stock was produced on Vero E6 cells at a multiplicity of infection of 0.01 in DMEM medium (Merck, Germany) supplemented with 10% of fetal bovine serum (Cytiva, USA). After 72h of incubation at 37°C, 5%CO_2_, the medium was centrifuged at 200g for 5min at 4°C to remove cellular debris. Supernatant was harvested, mixed with 5.750ml of Hepes (50mM) sucrose (500mM) solution and stored at -80°C as aliquots. Stock infectious titers were estimated by fluorescent focus assay on C6/36 cells (derived from *Ae. albopictus* larvae) (6). The day before infectious blood meal, fresh human blood (originating from multiple human anonymous donors, French National Blood Bank – Etablissement Français du Sang) was washed 3 times in PBS to recover erythrocytes. Mosquitoes were placed in feeding boxes (*i.e.* plastic containers covered with a net) then transferred at 26°C in the BSL3. The day of the infectious blood meal, CHIKV stock was mixed with the erythrocytes suspension (1:2, v:v) to reach an infectious titer of 10^7^ FFU.ml^-1^ in the blood meal. Blood feeding was performed with Hemotek feeders (Hemotek) covered with porc intestine and filled with 2.5mL of infectious blood heated at 37°C. Female mosquitoes were allowed to feed for 25 min. After blood meal, they were anesthetized, on ice, at 4°C for 30 min and fully engorged females were transferred in 1-pint cardboard cup (up to 25 females/cup) then incubated at 26.5°C, 16h/8h light/dark and 80% of humidity in presence of a cotton imbibed with a 10% sucrose solution. Aliquots of infectious blood were stored -80°C to control CHIKV infectious titer upon blood meal duration. Two independent blood meals were performed with two generation of parasitized and unparasitized females. The measured CHIKV titer measured at the end of each blood meal was 0.93x10^7^ FFU.ml^-1^ (experiment 1) and 1.15x10^7^ FFU.ml^-1^ (experiment 2).

Vector competence assay

Female mosquitoes were collected at 3, 7 or 14 days post infectious blood meal and processed to measure infection, dissemination and transmission rates by estimating CHIKV prevalence in mosquito body, head and saliva respectively. For each time point, individuals were anesthetized on ice. Their legs and wings were removed and they were glued on a plate with double sided tape (3M). Their proboscis was inserted in a 10µL filter tip containing 10µL of Fetal Bovine Serum (Cytiva) to collect saliva, as described (7). A 1.5 µL drop of 1% pilocarpine (Merck) supplemented with 0.1% Tween-20 (Sigma) was added on the thorax of each female to stimulate salivation in the filter tip. Salivations were performed for 1h at 26.5°C. Saliva solution transferred in a tube containing 100µL of culture medium (DMEM 1X with 4% FBS, 2.5µg/mL of Amphotericin, 100U/mL of Nystatin, 50µg/mL of Gentamicin, 100U/ml of Penicillin, 100µg/mL Streptomycin) and stored at -80°C. Heads and bodies of each individual were harvested and transferred in tubes containing 400µL of culture medium and a 3mm tungsten bead (Qiagen). Those tissues were crushed by agitation in a Qiagen Tissue Lyser II for 1min at 30Hz and stored at -80°C.

Estimation of CHIKV prevalence

CHIKV detection was performed on 96 well plates coated containing 3x10^5^ C6/36 cells and supplemented with 40µL of saliva, head or body samples. Plates were incubated for 3 days at 28°C. They were fixed with 150µL of 4% paraformaldehyde (Santa Cruz Biotechnology) for 20 min at room temperature. The overlay was removed and cells were washed three times with 100µL of DPBS (ThermoFisher scientific). Cell permeabilization was conducted by adding 50µL of a DPBS solution supplemented with 0.3% Triton-X 100 (Sigma) and 1% of Bovine Serum Albumin (BSA, Cytiva) for 30min at 37°C. Samples were washed three times with 100µL of DPBS. A volume of 50µL of a Semliki Forest virus anti-capsid antibody cross-reacting with CHIKV diluted 1:600 in DPBS and 1% BSA was added in each well for 1h at 37°C. This antibody was previously shown to be sensitive toward CHIKV (8). Cells were washed three time with 100µL of DPBS before being incubated for 1h at 37°C with 50µL of a 1:500 diluted goat anti-mouse secondary antibody coupled with an Alexa fluor 488 fluorochrome (Invitrogen). They were then washed three more times with DPBS before being observed under an epifluorescence microscope (Zeiss Colibri 7) at a 100X magnification. Samples for which the cell layers contained fluorescent foci were scored positive for CHIKV while those for which no fluorescent foci were observed were considered as uninfected. Positive (CHIKV stock) and negative (culture media) controls were performed on each titration plate. Infection, dissemination and transmission efficiencies corresponded to the prevalence of CHIKV (*i.e.* proportion of positive samples) in body, head and saliva respectively.

Quantification of CHIKV and *Ascogregarina taiwanensis*

Total RNA isolation was performed using 30µL of mosquito sample (either saliva, head or body) mixed with 70µL of TRIzol (Invitrogen). After 10 min at room temperature, 20µL of chloroform were added then vortexed and centrifuged for 15min at 17,000g, 4°C. The supernatant was collected and mixed with 60µL of isopropanol supplemented with 1µL of glycoblue (Invitrogen) before being homogenized and stored overnight at -20°C. Samples were then centrifuged for 15min at 17,000g and 4°C. Supernatant was discarded and the pellet was resuspended in 500µL of 70% cold ethanol before being pelleted by centrifugation for 15min at 17,000g and 4°C. After discarding the ethanol, RNA was dissolved in 10µL of RNAse free sterile water (Gibco). cDNA synthesis was performed with the iScript cDNA Synthesis kit (Bio-rad) following the manufacturers’ recommendations. qPCR quantification of CHIKV load was performed by targeting the *E2* envelope gene coding on CHIKV-positive head, body and saliva samples according to previous FFA titration. *As. taiwanensis* density was estimated by qPCR quantification of the *18S* ribosomal gene in mosquito bodies. For each samples (except saliva), viral gene copies were normalized with copy number of the mosquito housekeeping gene *rps17* coding a ribosomal protein. Specific forward and reverse primers for each gene were listed in **Table S3**. Briefly, quantifications were conducted in a 10µL volume with 1µM of each primer, 1X of Roche SYBR Green I master mix (Roche) and 2.5µL of 1/5^th^ cDNA dilutions. Amplifications were performed on a CFX 1000 thermocycler (Bio-rad) with 5min of denaturation at 95°C, followed by 45 cycles including 10s at 95°C, 30s at 50°C and 10s at 72°C. A fusion step was conducted with 5s at 95°C and a temperature gradient ranging from 65°C to 97°C. For E2 and rps17, 10 times serial dilutions of amplicons were used as a standard after purification with the QIAquick PCR purification kit (Qiagen) following manufacturers recommendations. For the parasite, 10 times serial dilutions of a PCR 2.1-TOPO TA vector (Invitrogen) with an insert of *As. taiwanensis* 18SrDNA was used as a standard.

Statistical analysis

Statistical analyses were conducted with the R software v.4.3.3.1. Prevalence data were modeled with a Generalized Linear Mixed Model using a binomial distribution and a logistic link function. Prevalence of the virus (*i.e.* presence – absence) was considered as a response variable while presence of the parasite, body part (including saliva), time post infection and their interactions were used as explanatory variables. Experiment replicates were used as a random variable. Influence of the explanatory variables on the viral prevalence was assessed with a Wald χ^2^ test followed by a *post-hoc* Tukey HSD test with the *car* and *emmeans* R packages respectively. Quantification data were modeled with a Linear Mixed Model (*i.e.* using a normal distribution). Quantification of the virus was considered as a response variable while presence of the parasite, body part (including saliva), time post infection and their interactions were used as explanatory variables. Experiment replicates were used as a random variable. Influence of the explanatory variables on the viral quantification was assessed with a type II ANOVA test followed by a *post-hoc* Tukey HSD test with the *car* and *emmeans* R packages respectively. Correlations between virus and parasite quantification values were conducted with a Spearman test.

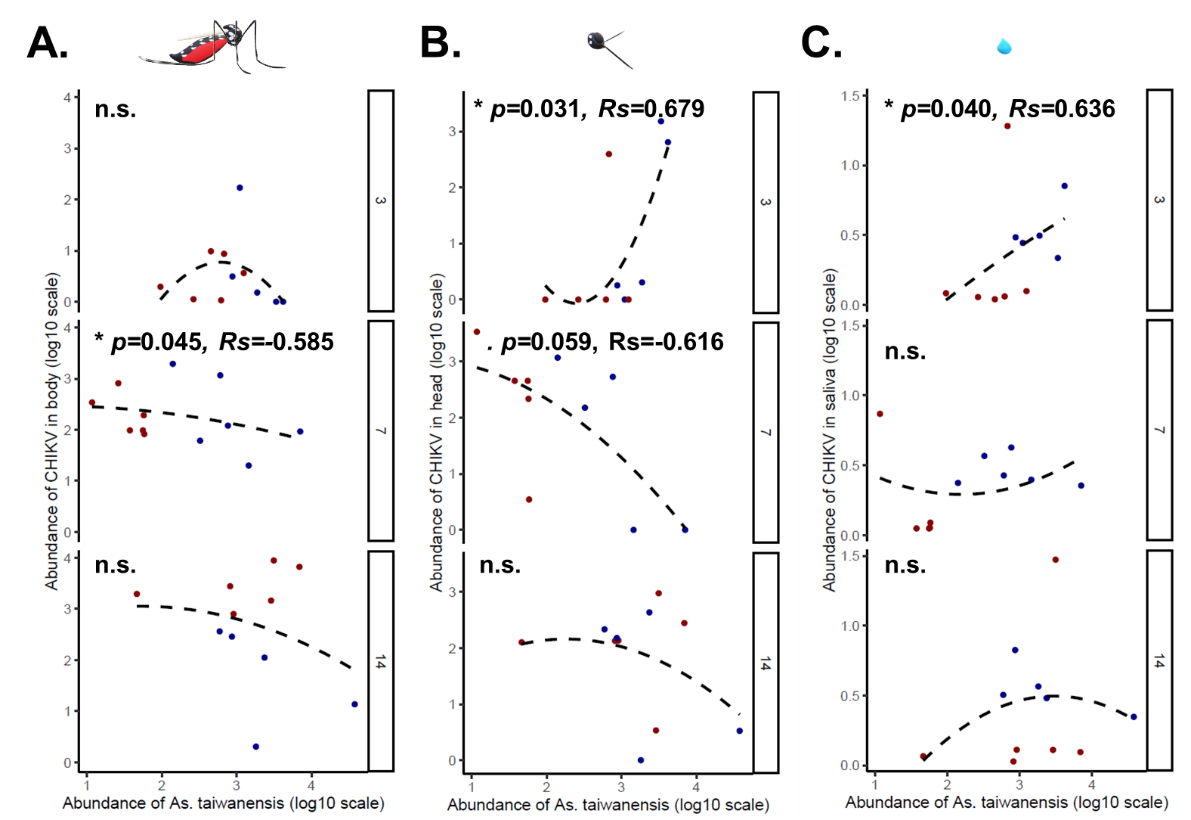


**Figure S1. Correlations between parasite and virus densities in co-infected individuals.** Densities of the parasite were correlated with those of the virus for each time point (3-, 7- and 14-days post infection) in mosquitoes (A) carcasses, (B) head and (C) saliva. Different dots colors represent different experiments. Non-linear Spearman correlation indices (Rs) and p-value (p) were represented for combinations of conditions showing either a significant correlation (*) or a tendency (.) i.e. p-value < 0.05 or <0.1 respectively. Non-significant (n.s.) correlations were annotated. Parasite densities are within the same range than those of field populations (1).

**Bibliography**

1. Girard M, Martin E, Vallon L, Tran Van V, Da Silva Carvalho C, Sack J, et al. Human-aided dispersal and population bottlenecks facilitate parasitism escape in the most invasive mosquito species. PNAS Nexus. 2024 May 1;3(5):pgae175.

2. Lindh E, Argentini C, Remoli ME, Fortuna C, Faggioni G, Benedetti E, et al. The Italian 2017 Outbreak Chikungunya Virus Belongs to an Emerging Aedes albopictus–Adapted Virus Cluster Introduced From the Indian Subcontinent. Open Forum Infect Dis. 2018 Dec 12;6(1):ofy321.

3. Rezza G, Nicoletti L, Angelini R, Romi R, Finarelli AC, Panning M, et al. Infection with chikungunya virus in Italy: an outbreak in a temperate region. Lancet. 2007 Dec 1;370(9602):1840–6.

4. Jourdain F, Valk H de, Noël H, Paty MC, L’Ambert G, Franke F, et al. Estimating chikungunya virus transmission parameters and vector control effectiveness highlights key factors to mitigate arboviral disease outbreaks. PLOS Neg Trop Dis. 2022 Mar 4;16(3):e0010244.

5. Martin E, Vallon L, Da Silva Carvalho C, Girard M, Minard G. Gregarine parasites are adapted to mosquito winter diapause. Parasites Vectors. 2022 Jul 11;15(1):249.

6. Viginier B, Cappuccio L, Garnier C, Martin E, Maisse C, Valiente Moro C, et al. Chikungunya intra-vector dynamics in *Aedes albopictus* from Lyon (France) upon exposure to a human viremia-like dose range reveals vector barrier’s permissiveness and supports local epidemic potential. Peer Commu J [Internet]. 2023 [cited 2023 Oct 16];3. Available from: https://peercommunityjournal.org/articles/10.24072/pcjournal.326/

7. Heitmann A, Jansen S, Lühken R, Leggewie M, Schmidt-Chanasit J, Tannich E. Forced Salivation As a Method to Analyze Vector Competence of Mosquitoes. J Vis Exp. 2018 Aug 7;(138):57980.

8. Greiser-Wilke I, Moennig V, Kaaden OR, Figueiredo LTM. Most Alphaviruses Share a Conserved Epitopic Region on Their Nucleocapsid Protein. J Gen Viro. 1989;70(3):743–8.
